# Toxicology Test Results for Public Health Surveillance of the Opioid Epidemic: Retrospective Analysis

**DOI:** 10.2196/50936

**Published:** 2023-09-28

**Authors:** Titus Schleyer, Bill Robinson, Samir Parmar, Diane Janowiak, P Joseph Gibson, Val Spangler

**Affiliations:** 1 Center for Biomedical Informatics Regenstrief Institute, Inc Indianapolis, IN United States; 2 School of Medicine Indiana University Indianapolis, IN United States; 3 hc1 Insights, Inc Indianapolis, IN United States; 4 Atlanta, GA United States; 5 Marion County Public Health Department Indianapolis, GA United States

**Keywords:** opioid epidemic, clinical laboratory techniques, public health, epidemiology, toxicology

## Abstract

**Background:**

Addressing the opioid epidemic requires timely insights into population-level factors, such as trends in prevalence of legal and illegal substances, overdoses, and deaths.

**Objective:**

This study aimed to examine whether toxicology test results of living individuals from a variety of sources could be useful in surveilling the opioid epidemic.

**Methods:**

A retrospective analysis standardized, merged, and linked toxicology results from 24 laboratories in Marion County, Indiana, United States, from September 1, 2018, to August 31, 2019. The data set consisted of 33,787 Marion County residents and their 746,681 results. We related the data to general Marion County demographics and compared alerts generated by toxicology results to opioid overdose–related emergency department visits. Nineteen domain experts helped prototype analytical visualizations. Main outcome measures included test positivity in the county and by ZIP code; selected demographics of individuals with toxicology results; and correlation of toxicology results with opioid overdose–related emergency department visits.

**Results:**

Four percent of Marion County residents had at least 1 toxicology result. Test positivity rates ranged from 3% to 19% across ZIP codes. Males were underrepresented in the data set. Age distribution resembled that of Marion County. Alerts for opioid toxicology results were not correlated with opioid overdose–related emergency department visits.

**Conclusions:**

Analyzing toxicology results at scale was impeded by varying data formats, completeness, and representativeness; changes in data feeds; and patient matching difficulties. In this study, toxicology results did not predict spikes in opioid overdoses. Larger, more rigorous and well-controlled studies are needed to assess the utility of toxicology tests in predicting opioid overdose spikes.

## Introduction

A key challenge in addressing the opioid epidemic [1,2] is timely insight into population-level factors, such as trends in prevalence of legal and illegal substances, overdoses, and deaths. Many surveillance systems and approaches at the national, regional, and local levels exist [3-12] but are limited by being (1) focused on late-stage outcomes such as drug-related arrests and overdose deaths [13,14], (2) frequently not available until long after an event occurs [15,16], (3) drawn from fragmented and siloed data, and (4) not representative [17].

This project explored whether toxicology laboratory results [18,19] from testing in health care and jail settings (in short, “toxicology results”) are potentially useful in surveilling the opioid epidemic. Our work builds on similar efforts to leverage calls to poison control centers for surveillance [5]. These selected toxicology tests may be useful because they occur in a variety of settings where effects of changing opioid use may first become apparent, can be communicated in real time through health information technology standards such as Health Level 7 (HL7) [20] and Logical Observation Identifiers Names and Codes (LOINC) [21,22], can be integrated at an individual level using record linkage [23], and are required in many states' prescription drug monitoring programs and are recommended for monitoring patients on chronic opioid therapy [24,25].

To date, toxicology results have been used primarily for retrospective, one-off analyses [26-29]. The goal of this project was to investigate whether ongoing, timely monitoring of living individuals' toxicology results gathered from several sources might indicate changes in the general population's opioid use. This study addresses the following questions: (1) how can toxicology test data from multiple sources be aggregated and homogenized? (2) What are the characteristics of persons in an aggregated set of toxicology test data in Marion County, Indiana, United States, compared to those of the general population? (3) Can toxicology test data provide direct indicators for trends regarding the opioid epidemic? (4) How might such toxicology test data be integrated into a dashboard for managing the opioid epidemic?

## Methods

### Overview

The health care company managing this project receives many types of laboratory tests from its clients, which are primarily clinical laboratories. The orders for and results of these tests are transmitted to the health care company in near real time using the HL7 protocol. The transmissions typically contain patient demographics, ordering provider and location, specimen information, the ordered tests, and the quantitative or qualitative results. All laboratory test information is transmitted to a data lake where it is refined, enriched, and deidentified.

### Sample Characteristics

The data set for this study included all toxicology results with a patient (or, if not available, an order or accession) address in Marion County (the largest county in Indiana with a population of 954,760 individuals as of 2018; home to Indianapolis—the capital of Indiana) collected between September 1, 2018, and August 31, 2019.

Patient and provider records were refined using several methods, including standardizing variable values and formatting, decomposing composite fields, and retrieving missing information (eg, ZIP codes based on address). We refined organizations through a similar process and categorized them by type, such as addiction treatment centers, criminal justice, forensics, hospital, emergency department, pain management, and primary care. Patient records were linked through a string similarity function that assigned a master patient identifier if records had a match rate of 95% or greater.

Matching tests across multiple laboratories was one of the most challenging aspects of cleaning and homogenizing the data. We used string matching functions and manual review to assign a LOINC code to each test, which we then mapped to a local drug class hierarchy.

For result records, abnormality was calculated by comparing the result value with the transmitted reference range. Positivity for toxicology tests, based on keywords or numeric values, were determined by profiling client data. Multiple tests for the same patient were considered as separate, with the exception of pairs of screening and confirmatory tests (which occurred rarely and were considered positive if the confirmatory test yielded a positive finding).

We only included records with ZIP codes from Marion County (either the patients’ or, if unavailable, the ordering location’s records). We retained only the data from hospitals, primary care providers, clinical specialty providers, and jails because emergency department, coroner, forensics, police department, sheriff’s offices, state police, and employer testing data were expected to exhibit markedly different result patterns. For instance, in emergency departments and law enforcement, sampling due to suspected alcohol and drug use typically results in high positivity rates. Positivity rates for employment drug testing, on the other hand, are often low since individuals applying for jobs know a drug test is required. Because these patterns were observed in our data, we excluded results from these settings. We included data from jails because positivity rates were fairly consistent with those reported in Marion County.

In total, 24 clients of the health care company had data for at least 1 Marion County patient. The largest contributor provided 64% of the results’ volume but only supplied data from January to April 2018. We excluded these data because they mostly comprised employment testing and had, comparatively, a much lower positivity rate. Of the remaining data, 67% of them were obtained from a regional reference laboratory and the core laboratory for several hospitals in Indiana, and the next 15% of them were obtained from a laboratory carrying out testing for law enforcement and forensics (only jail data were included). The remainder of the laboratories were primarily regional toxicology and reference laboratories. In addition to our data refinement and linking infrastructure, we already had built a preliminary dashboard for visualizing the data that served as the basis for this project [30].

### Dashboard Development and Data Analysis

The project was advised by a 9-member external advisory group consisting of 3 academic researchers; 5 public health professionals at the local, state, and international level; and 1 corporate participant. This group met several times with 8 health care company staff members and executive leaders over the course of the project period to provide high-level strategic guidance. A technical working group, consisting of 3 members of the external advisory group and company technical personnel, prepared and analyzed the data and designed and prototyped the dashboard.

After data preparation, we summarized toxicology results data descriptively and compared them to data for Marion County where possible. We performed 2-proportion *z* tests on each category, excluding unknown counts in totals. In addition, we developed a set of design ideas for a local dashboard to manage the opioid epidemic and evaluated them through a survey of the advisory group and additional company personnel. In the survey, we presented proposed design features and asked one or more questions, such as “What kind of useful information can you glean from the presented visualization?” “What kind of information is missing?” “Is it easy to determine values of interest?” The survey was distributed to 19 invitees (8 advisory group members and 11 company staff).

Last, we evaluated how toxicology results trends related to signals derived from opioid overdose–related emergency department visits. The goal of this analysis was to determine whether simple positivity rates from toxicology results can provide useful signals for trends regarding the opioid epidemic. For instance, intuition would suggest that test positivity rates might rise prior to spikes in overdoses. For the toxicology tests, we used the specimen collection date, and for emergency department encounters, the visit date.

The Marion County Department of Health uses ESSENCE (Electronic Surveillance System for the Early Notification of Community-Based Epidemics) [31] to analyze opioid-related data, such as opioid overdose–related emergency department visits [32], and generate alerts for notable events. To detect spikes in test positivity, or the incidence of opioid overdose–related emergency department visits, we applied the ESSENCE C2 detection method to toxicology results and emergency department opioid overdose data between September 1, 2018, and August 31, 2019. The algorithm uses a moving sample average and sample SD to standardize each observation, with a 2-day lag in the mean and SD calculations [33]. The implemented baseline in ESSENCE is 28 days, compared to the baseline of 7 days. For emergency department data, the opioid outbreak indicator was the daily count of individuals with any overdose, and for laboratory data, the daily positive proportion of opioid toxicology tests. If the result exceeded 3 SDs above the sample mean, an alert was generated.

### Ethics Approval

This project (protocol #1802267756: Development and formative evaluation of the Opioid Epidemic Management Dashboard) was approved as expedited by the Indiana University institutional review board on February 2, 2018.

## Results

### Overview

Table 1 shows a comparison of the major characteristics of the health care company’s data set and Marion County demographics. For the study period, 4% of people with a Marion County address had at least 1 test result. The health care company data set’s gender distribution (35.9% males and 64.1% females—within the 82.5% of individuals with a known gender) differed significantly from the gender distribution of Marion County’s population (48.2% males and 51.8% females). A much larger, national data set of test results had a more similar gender distribution (40.5% males and 59.5% females). In numerous records, data on race and ethnicity were missing and therefore not included. Age distributions (within the 81.9% of individuals with a known age) also showed differences, with individuals aged up to 19 years significantly underrepresented and those aged 20 to 39 years significantly overrepresented in the health care company data set.

Table 2 provides additional detail about toxicology results for the 37 ZIP codes in Marion County. The proportion of residents by ZIP code with at least 1 toxicology test result within the study period ranged from 0.4% to 41.5%. In 28 (76%) ZIP codes, the range was between 0.4% and 3%; in 5 (14%), between 5.1% and 8.4%; and in 4 (11%), between 10.7% and 41.5%. For the 46.2% of records having no patient address, the ZIP code of the ordering location was used, which implies that ZIP codes with large order volumes and low populations showed higher percentages of tested residents. For example, ZIP code 46202, which has the highest percentage at 41.5%, is the location of Marion County Jail II. The next 4 highest percentages are in ZIP codes that include major hospitals. The result positivity rate, defined as the number of positive results divided by the number of nonmissing or nondeterminate results, ranged from 3% to 19%. Visits to the emergency department due to overdose and overdose deaths are provided for context. However, it should be noted that the time periods for the number of residents and overdose deaths are for 2018, only partially overlapping with the September 2018 to August 2019 date range of the laboratory tests.

**Table 1:** Comparison of gender and age characteristics of the health care company’s data set (September 1, 2018, to August 31, 2019) to Marion County demographics obtained from US Census Bureau (2018).

		Health care Company (N=33,787), n (%)	Marion County (N=954,670), n (%)	*P* value (*Z* test)
**Gender**				
	Male	10,012 (35.9)	460,093 (48.2)	<.01
	Female	17,856 (64.1)	494,577 (51.8)	<.01
	Unknown	5923 (N/A^a^)	N/A (N/A)	N/A
**Age (years)**				
	0-19	3901 (14.1)	257,636 (27.0)	<.01
	20-39	14,268 (51.5)	293,706 (30.8)	<.01
	40-59	6046 (21.8)	228,542 (23.9)	<.01
	60-79	3088 (11.2)	145,891 (15.3)	<.01
	>80	391 (1.4)	28,895 (3)	<.01
	unknown	6093 (N/A)	N/A (N/A)	N/A

^a^N/A: not applicable.

**Table 2:** Toxicology results for the 37 ZIP codes in Marion County, Indiana, United States, sorted by the number of residents in descending order from September 1, 2018, to August 31, 2019 (except for the number of residents and overdose deaths from January 1, 2018, to December 31, 2018).

ZIP code	Residents, n	Residents in the data set, n (%)	Residents with positive results, n	Results, n	Positive results, n	Result positivity rate (%)	Overdose-related emergency department visits, n	Overdose deaths, n
46227	56,449	2931 (5.2)	1545	43,211	4508	10.4	264	20
46226	45,998	1183 (2.6)	729	18,119	1842	10.2	208	14
46237	39,803	427 (1.1)	268	7312	934	12.8	128	13
46203	38,313	546 (1.4)	359	7958	1023	12.9	282	20
46254	36,530	934 (2.6)	424	17,121	1238	7.2	66	8
46224	35,177	575 (1.6)	331	9977	1382	13.9	93	<5
46220	33,833	751 (2.2)	439	11,509	1423	12.4	64	<5
46219	33,646	4407 (13.1)	3030	59,509	8048	13.5	269	17
46222	33,061	372 (1.1)	219	4774	534	11.2	153	19
46260	32,779	2768 (8.4)	1690	40,864	4746	11.6	79	<5
46241	30,918	420 (1.4)	288	3488	550	15.8	177	23
46218	30,516	903 (3.0)	587	12,892	1306	10.1	215	16
46201	30,487	863 (2.8)	639	13,216	1817	13.7	361	34
46217	29,558	296 (1.0)	165	4082	363	8.9	103	10
46235	29,507	766 (2.6)	387	11,117	817	7.3	99	5
46229	27,913	1437 (5.1)	1019	21,806	4182	19.2	105	13
46221	27,054	206 (0.8)	105	2988	295	9.9	146	7
46236	26,751	462 (1.7)	242	6522	546	8.4	74	<5
46268	26,411	602 (2.3)	342	8511	726	8.5	64	<5
46205	26,351	553 (2.1)	293	8037	668	8.3	86	11
46234	25,002	158 (0.6)	77	2299	180	7.8	34	7
46239	24,348	311 (1.3)	134	4594	304	6.6	91	5
46214	23,747	275 (1.2)	108	3984	243	6.1	46	<5
46256	23,541	3887 (16.5)	1847	54,212	4155	7.7	55	5
46208	23,312	290 (1.2)	181	4042	394	9.7	77	7
46240	18,817	374 (2.0)	230	4912	478	9.7	40	<5
46250	18,545	490 (2.6)	282	7271	683	9.4	28	5
46202	16,021	6652 (41.5)	5447	263,328	19,823	7.5	139	5
46113	15,037	72 (0.5)	27	1048	66	6.3	22	<5
46228	14,876	182 (1.2)	104	2570	220	8.6	36	<5
46107	12,801	110 (0.9)	49	1721	122	3.0	70	5
46259	11,777	61 (0.5)	25	782	62	7.9	30	<5
46231	11,440	47 (0.4)	24	681	68	10.0	19	<5
46278	7968	98 (1.2)	50	1509	102	6.8	7	<5
46225	6524	489 (7.5)	417	66,361	2014	3.0	65	8
46204	5903	369 (6.3)	291	37,083	1066	2.9	82	<5
46216	1496	160 (10.7)	97	2348	297	12.6	<5	<5
Total	932,210	35,427 (3.8)	22,491	771,758	67,225	8.7	N/A^a^	N/A

^a^N/A: not applicable.

### Dashboard Design and Prototyping

We used the data to explore potential visualizations based on the dashboard we had previously developed [30]. We focused our efforts on designing specific enhancements to the dashboard and identifying potential improvements through a survey. Of the 19 invitees, 10 responded. A description of the main design enhancements and potential improvements follows.

**Figure 1:**
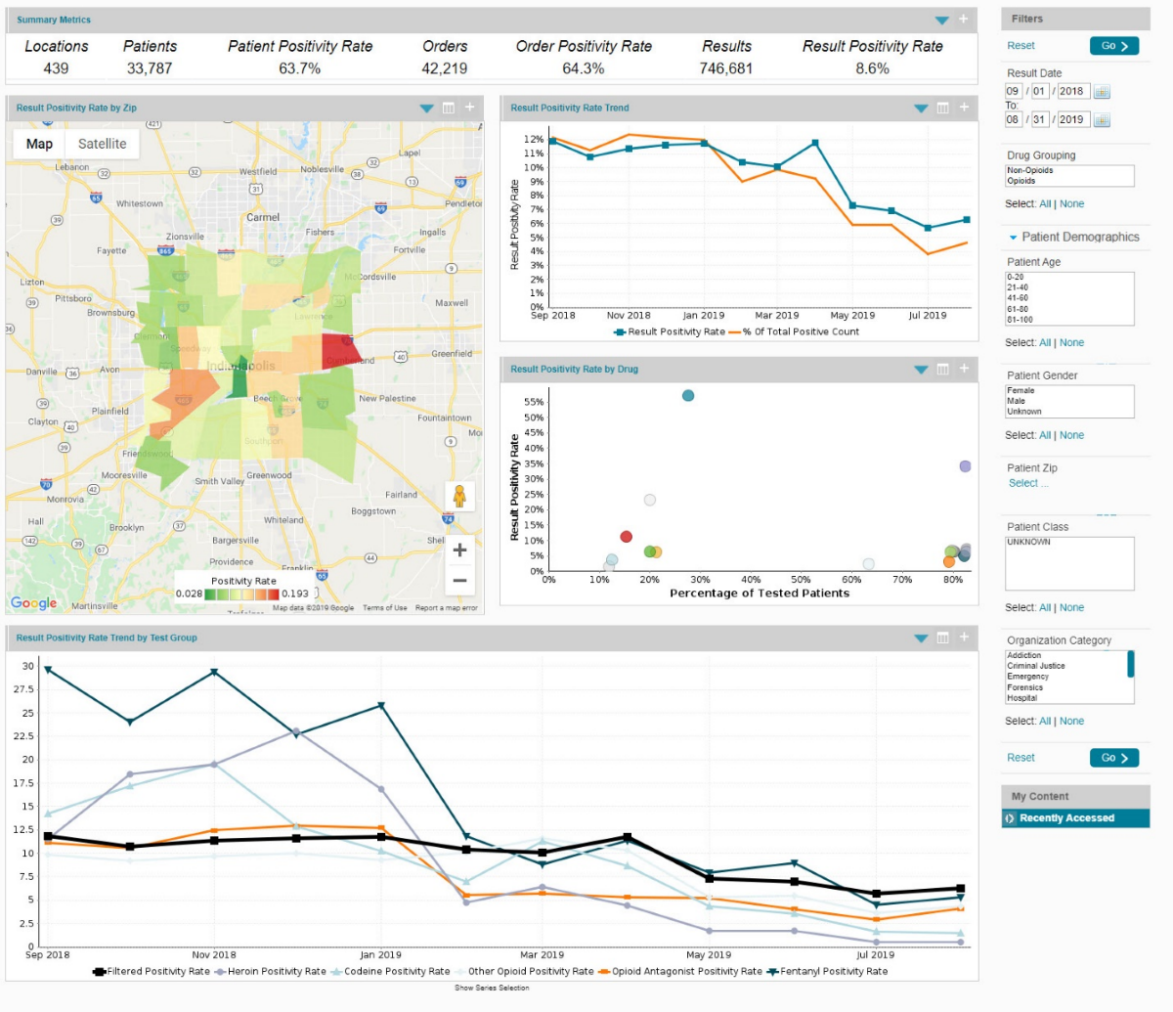
Final design of main dashboard displaying summary toxicology results for Marion County, Indiana, United States.

Figure 1 shows the final design of the main dashboard. The Summary Metrics bar near the top summarizes the data in general with regard to patients, test results, and positivity. The result positivity rate by ZIP heat map provides a geographic overview of Marion County; other graphs display general and drug-specific positivity trends and information. More information can be displayed by hovering over certain areas of the screen. Filters on the right allow the user to subset the data. The following sections provide additional detail and survey results for selected aspects of the design.

### Summary Metrics Display

The Summary Metrics display in Figure 1 shows the following key numbers:

- Locations: count of distinct physical ordering sites such as a doctor’s office, emergency department, employer, or clinic
- Patients: total number of individual patients with 1 or more test results
- Patient positivity rate: number of patients with at least 1 positive result divided by the number of patients with 1 or more test results
- Orders: total number of unique laboratory orders including 1 or more results
- Order positivity rate: the number of orders with 1 or more positive result divided by the number of orders with 1 or more results
- Results: total number of results (screening and confirmatory tests for the same drug only counted once)
- Result positivity rate: number of positive results divided by the total number of results

Feedback on the Summary Metrics display included the need for clearer labeling of selected metrics, separating toxicology results for licit or illicit substances, and adding contextual data, such as the total population from which the tests are drawn, naloxone administrations, fatal or nonfatal overdoses, overdose-related emergency medical services runs, prescriptions, and medication-assisted treatment volume.

### Result Positivity Rate by ZIP Code Display

Geographical maps are common in displaying data related to the opioid epidemic [34]. The result positivity rate by ZIP display in Figure 1 shows the map displaying test positivity rates across ZIP codes. Positivity rates range from 0.028 to 0.193. The user can pan and zoom in or out of the map, as well as select data to display using the filters on the right of the dashboard.

Feedback on this design included its usefulness for identifying “hot spots,” and the need to standardize the color range across displays with different minima and maxima of the positivity rate; providing the numerator and denominator for the positivity rate, as well as residents by ZIP code, to judge representativeness of the data; the ability to “scrub” through the time line; and the ability to correlate with other data, such as overdoses or emergency medical service runs. In addition, map areas did not correspond exactly with ZIP codes.

### Drug Positivity by Age, Gender, and Drug

**Figure 2:**
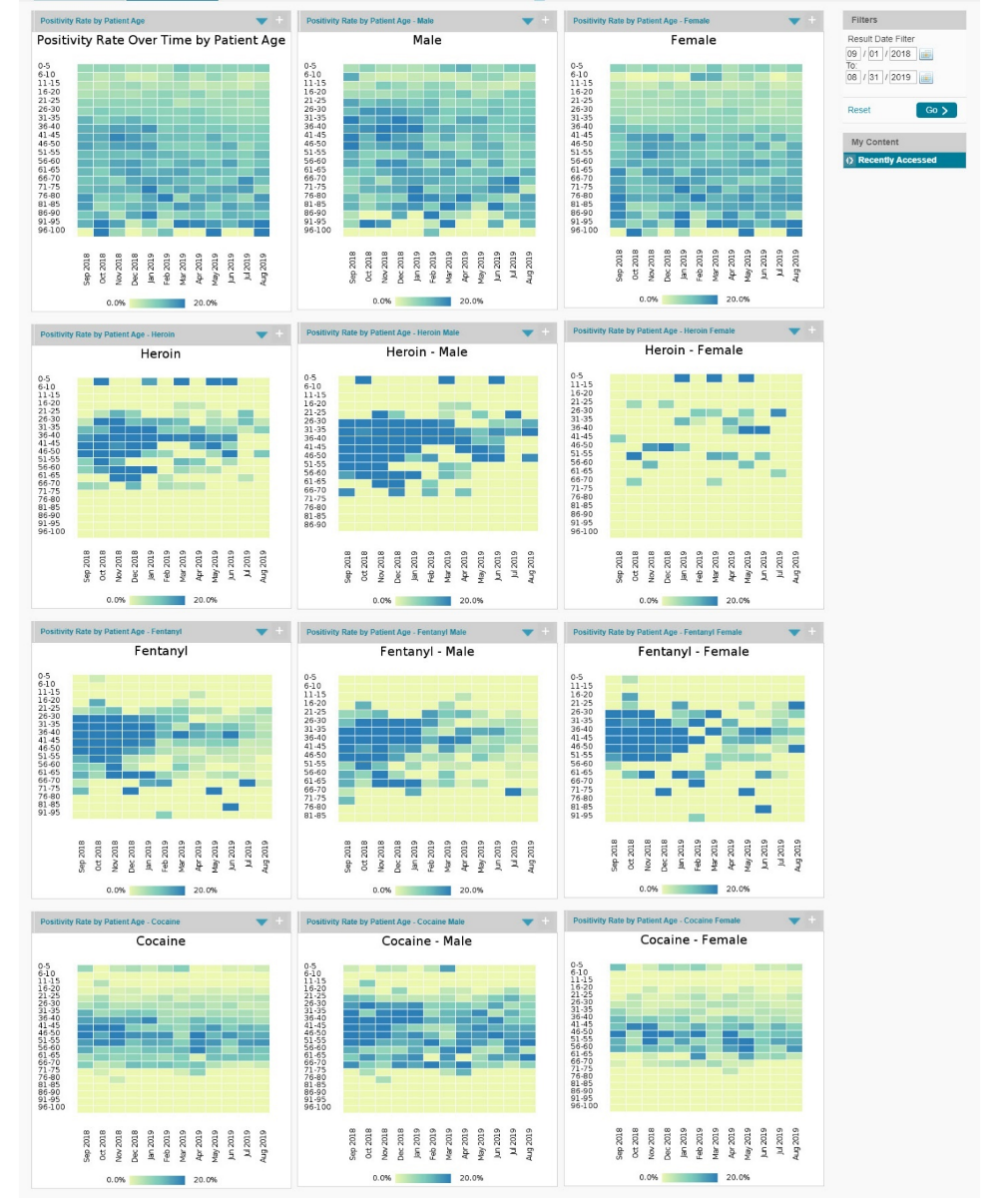
Visualizations for drug positivity by age, gender, and specific drug for Marion County, Indiana, United States. Data are from September 1, 2018, to August 31, 2019.

Figure 2 shows drug positivity by age, gender, and drug to understand multivariate relationships in the data. Certain patterns are evident, such as generally lower positivity rates for heroin in females than in males, and age and positivity differentials regarding cocaine.

Feedback on this design included that it was easy to tell which groups are high-risk and whether these groups were stable over time. It was perceived as difficult to tell how important or statistically significant the differences were between rectangles of different colors. An alternative design suggestion was a bar graph by age as an initial visual, with a drill-down option to look at time trends.

### Toxicology Results as Predictive Signals

We used the ESSENCE platform to determine the potential relationship between changes in toxicology results’ positivity and opioid-related chief complaints in emergency departments. We included toxicology results from our data set likely to be predictive for future overdoses, such as those generated in health care and jail facilities. We excluded data that were likely to have been collected after overdoses, such as emergency department and coroner data. During the 12 months of overlap between the data sets, ESSENCE generated 4 alerts for emergency department visits and 3 for toxicology results. We counted the combination of 1 alert each as an “episode” if (1) the toxicology result alert occurred prior to the emergency department visit alert and (2) both alerts occurred within a 30-day window but not on the same day. We chose 30 days as the time window because we considered toxicology results alerts outside of that window as not actionable. Only 1 episode occurred in the data set, with the toxicology results alert preceding the emergency department alert by 9 days. The sample size was not sufficient to conduct statistical tests for comparison.

## Discussion

The purpose of this project was to elucidate whether analyzing toxicology results may be useful in monitoring the opioid epidemic. Our key findings are summarized below.

### Challenges in Aggregating, Preparing, and Managing Toxicology Test Data From Multiple Sources

We aggregated data from 24 health care company clients, all of whom sent us data in varying formats and degrees of completeness. Attempts to combine data sources for population surveillance need to account for differing formats, rates of completeness, and missingness.

Changes in the client base and data feeds affected data availability. Where possible, we imputed missing data by matching to a more complete version of the patient record from another client, or using addresses of ordering providers and accessioning location as proxies. However, such imputations carried the risk of introducing bias.

Linking data was relatively easy because data were fully identified and could be matched across clients. However, we could not link toxicology results to external data such as nonfatal overdoses, overdose deaths, or naloxone administrations due to privacy constraints. This limitation reduced our ability to develop a more complete picture of the epidemic.

### Factors Increasing the Effort Required to Clean and Synthesize Data

Test names are often not standardized among laboratories, requiring significant computational or manual inferencing. While initial test mapping took considerable effort, we partially automated the process as the set of test names mapped to the hierarchy grew. Artificial intelligence methods using the training data generated in this project may facilitate test mapping in the future.

Toxicology test orders often include component tests for multiple drugs. The component tests, in turn, can have multiple instances such as screening (qualitative) and confirmatory (quantitative) tests for the same drug. Many confirmatory tests are a collection of metabolites that can indicate 1 or more parent drugs. We counted 1 or more positive results for the same drug within the same test order as a single result.

Toxicology results can sometimes be difficult to interpret with respect to the source substances introduced into the patient’s system and the metabolites detected at various time points.

### Representativeness of Toxicology Test Data for Larger Trends in the Opioid Epidemic

Toxicology tests are typically not administered to a random sample of the population. For instance, pain management patients are more likely to be tested when drug testing is required for chronic opioid therapy. Such consistency testing necessarily reflects the expectations of the clinician, such as a positive result when the patient is on opioid therapy. On the other hand, drug screening related to employment or Department of Transportation monitoring samples a different demographic with the expectation that most test results are negative. Inclusion or exclusion of data sets generated for various purposes will likely skew positivity rates. This may be partially addressed by only including data sources that are not likely to be strongly biased with regard to the test result, and weighting included data sources according to their demographic composition, to approximate the demographics of the population of interest. Encouragingly, the gender distribution in our results resembled that found in the results of a large, national laboratory test provider, providing some evidence of external validity. Unfortunately, demographic information is often missing in laboratory test records.

### Potential Approaches to Visualizing Toxicology Test Data

Our project generated several potentially useful ideas for visualizing toxicology test data. Summary statistics that include unique individuals, the number of orders and tests, and positive or negative test results for various analytes could help monitor drug use or abuse prior to serious events, such as overdoses and overdose deaths. A variety of visualization techniques can help show relationships among and trends for selected variables.

However, limitations in being able to integrate and interlink different data sets was a key obstacle for generating insights. For instance, several of our organizational participants had access to highly relevant data, such as prescriptions, fatal and nonfatal overdoses, emergency medical service runs, and drug seizures related to opioids. Interlinking these data on an individual basis (where possible) was perceived as potentially useful but challenging with regard to governance, record linking, and time and effort required.

### Using Toxicology Test Data as a “Signal” in Surveillance

Our results are inconclusive regarding the question of whether surveillance of toxicology results at the urban county level can serve as an effective predictor for spikes in opioid overdose cases admitted to emergency departments. While we focused on data likely to be predictive for such events, the proportion of overdose cases for which a toxicology test result is available prior to or after an overdose is unknown. In addition, chief complaints and discharge diagnoses for Marion County vary considerably by hospital with regard to specificity about overdoses and specific drugs involved. Individual-level data linkage may be a promising option to answer such questions and elicit more meaningful signals than possible in our study.

### Scaling Our Approach to Other Municipalities and States for Public Health Surveillance Purposes

Due to variation in data set content, availability, granularity, and linkability, our approach is likely difficult to scale easily to other municipalities and states. The health care company’s market position in Indiana provided a strong foundation for attempting to explore the utility of test results for tracking the opioid epidemic. However, even given that, it is unknown to what degree toxicology results in Marion County are indicative of trends in the opioid epidemic.

Currently, toxicology tests among living individuals appear to play only a small role in surveillance of the opioid epidemic. However, such tests might be important and timely indicators for drug use disorder trends in the general population. Further work should address issues identified in our study, such as aggregating, preparing, and managing toxicology test data; representativeness of these data; potential approaches to visualizing them; and using toxicology test data as a “signal” in surveillance.

### Conclusions

Analyzing toxicology results of living individuals from a variety of sources may be useful as an indicator of trends in opioid use. Important findings to consider include the following: (1) there are multiple challenges in aggregating, preparing, and managing toxicology test results for population trend analysis; (2) the representativeness of these data for the general population must be assessed carefully; (3) leveraging toxicology test results as a “signal” in surveillance likely requires robust data sets and sophisticated analyses. Individual-level data linkage may be a promising option to elicit more meaningful signals than is currently possible; and (4) a variety of visualization techniques can help show relationships among and trends for selected variables.

## Abbreviations

ESSENCE: Electronic Surveillance System for the Early Notification of Community-Based Epidemics
HL7: Health Level 7
LOINC: Logical Observation Identifiers Names and Codes
